# Supplementary figures and images for: Plant mobile domain proteins ensure Microrchidia 1 expression to fulfill transposon silencing
Source: Life Sci Alliance. 2023 Feb 2;6(4):e202201539. doi: 10.26508/lsa.202201539 (PMC9899485; doi:10.26508/lsa.202201539)

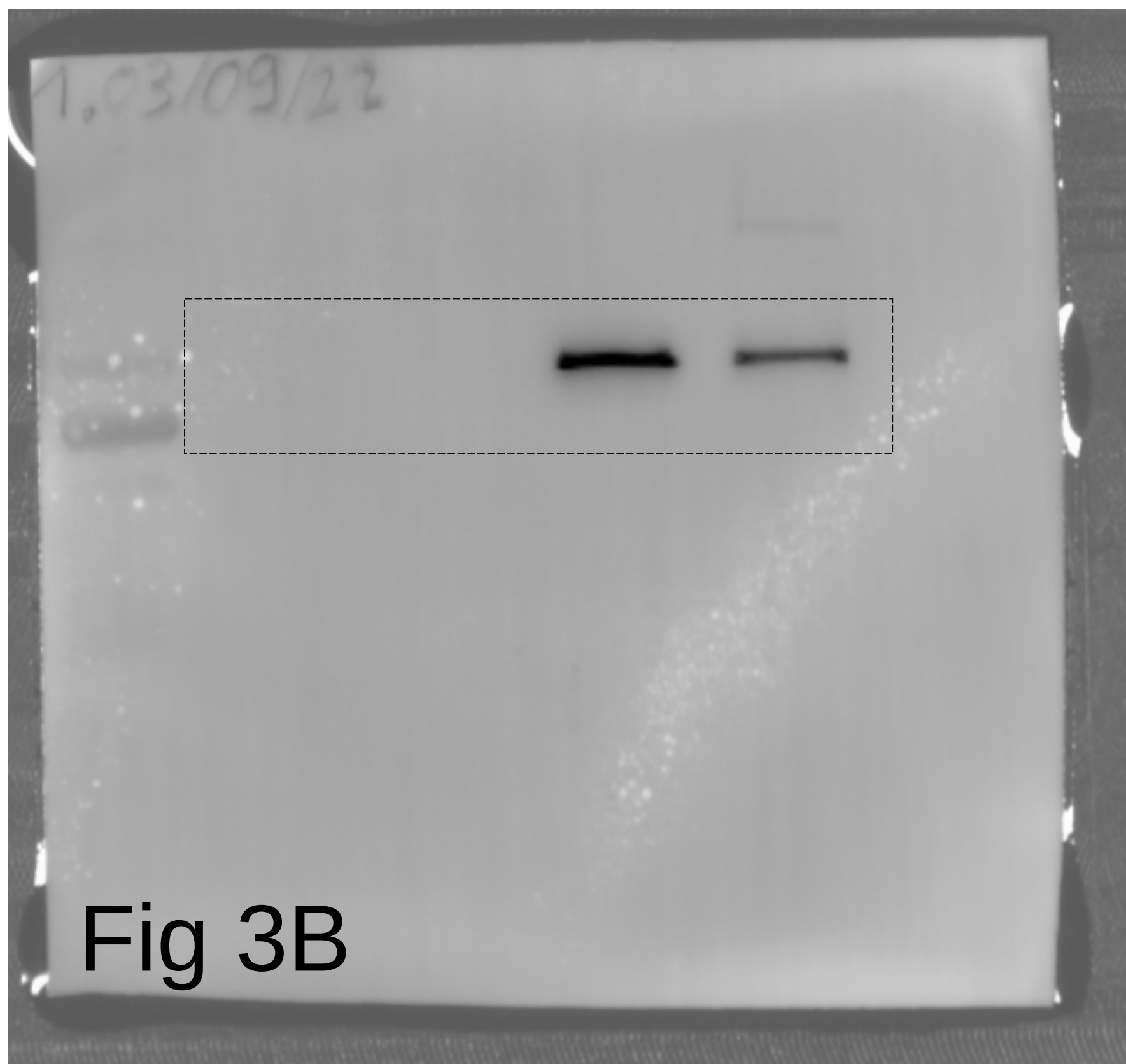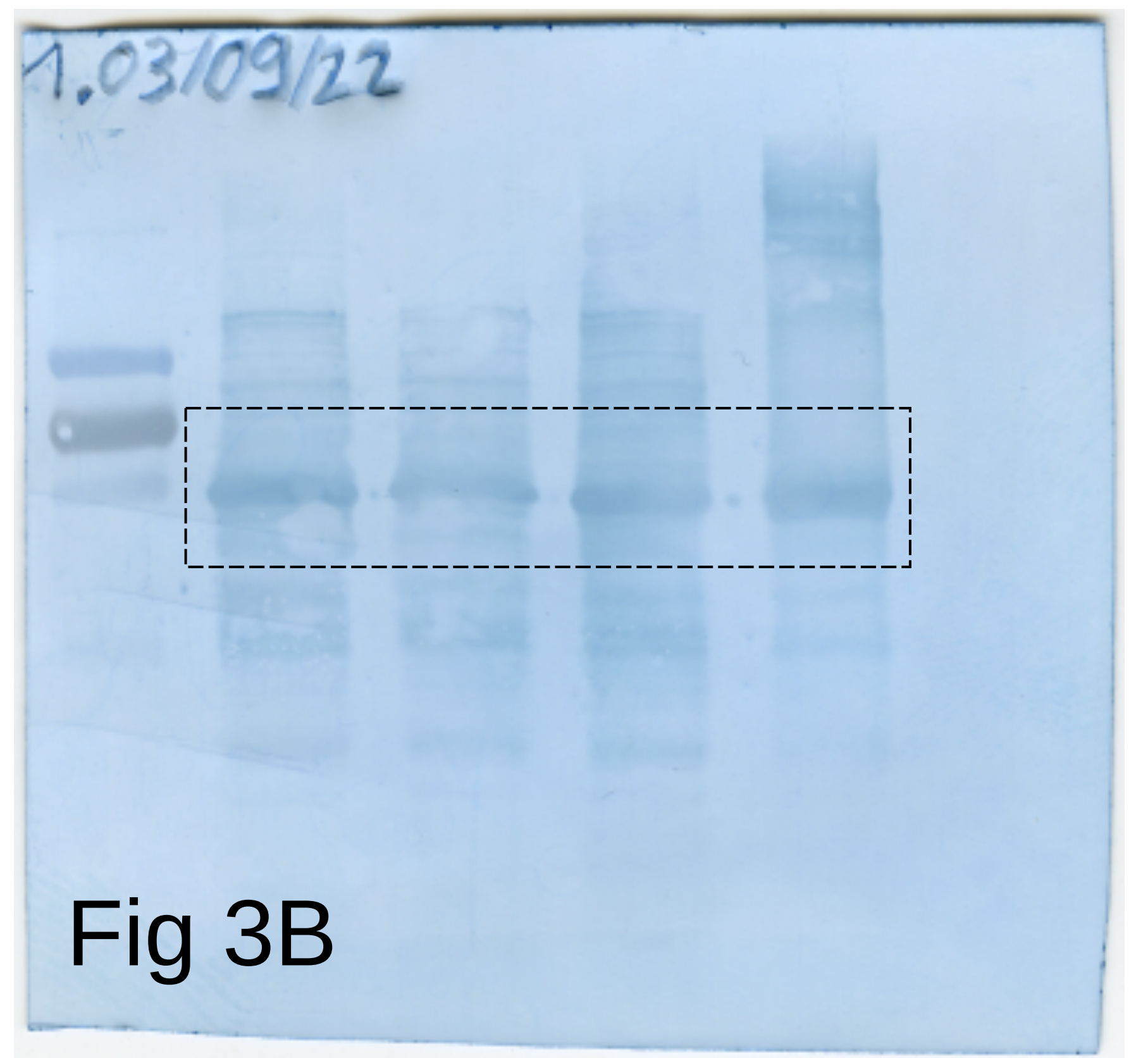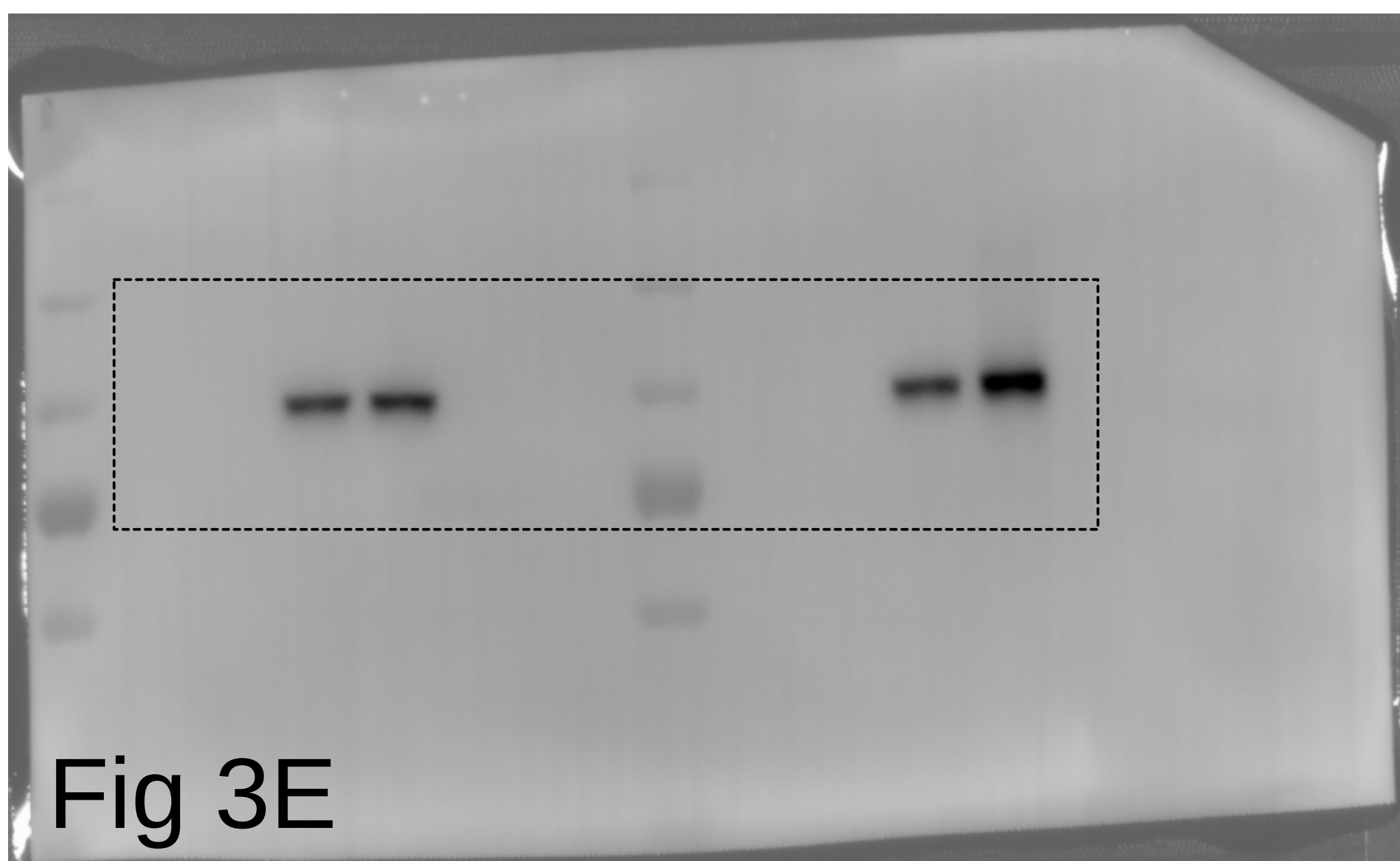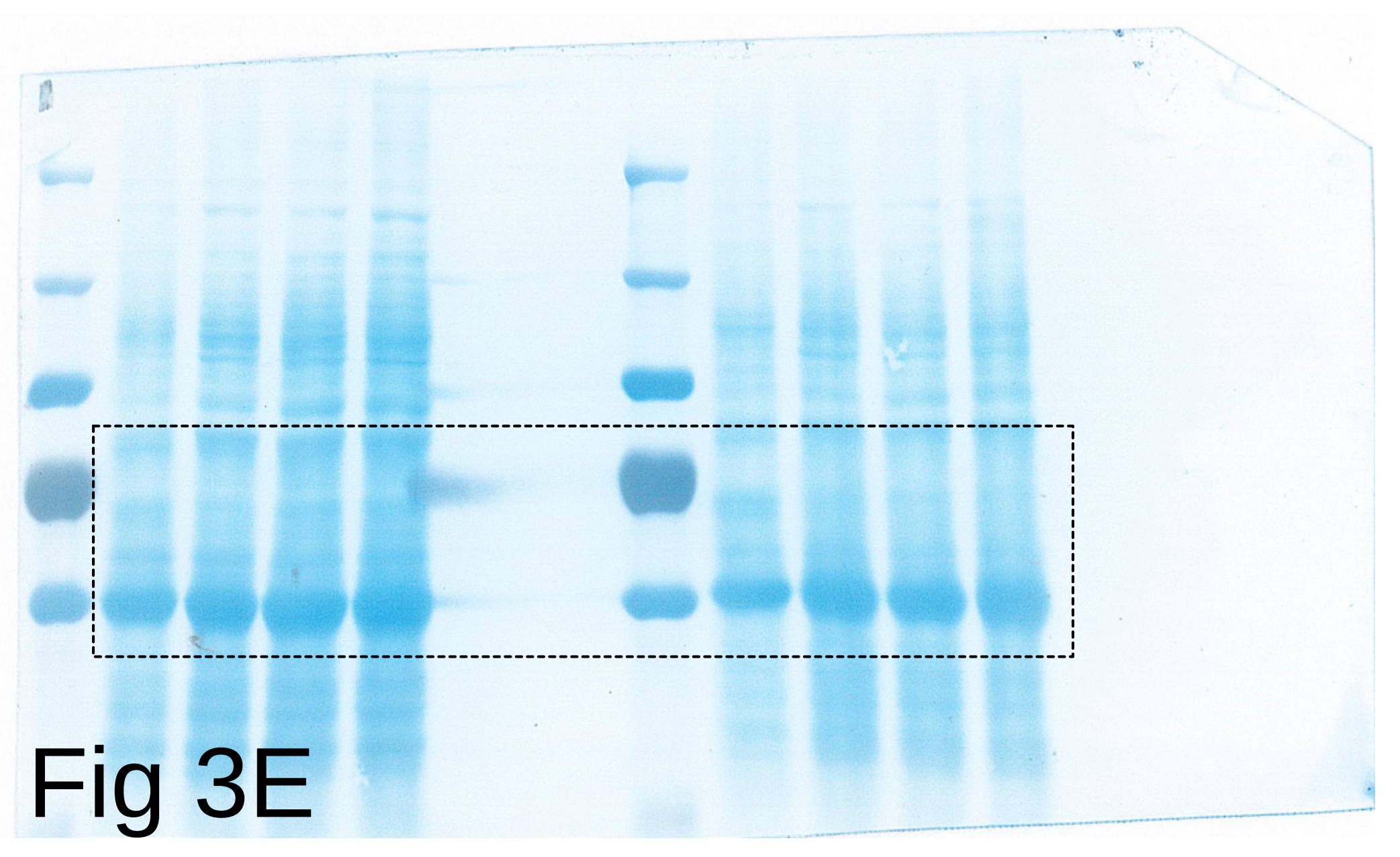

Supplement: Supplementary file 6 [file LSA-2022-01539_SdataF3.1.pdf]
